# Association of adverse childhood experiences and neurodevelopmental disorders in people with fetal alcohol spectrum disorders (FASD) and non-FASD controls

**DOI:** 10.1186/s12887-019-1878-8

**Published:** 2019-12-16

**Authors:** Cassondra Kambeitz, Marilyn G. Klug, Jacob Greenmyer, Svetlana Popova, Larry Burd

**Affiliations:** 1 Department of Pediatrics, University of North Dakota School of Medicine and Health Sciences, 1301 N Columbia Rd Stop 9037, Grand Forks, ND 58202-9037 USA; 2 Department of Population Health, University of North Dakota School of Medicine and Health Sciences, Grand Forks, ND USA; 3 Institute for Mental Health Policy Research, Centre for Addiction and Mental Health, Dalla Lana School of Public Health, University of Toronto, Toronto, Ontario Canada; 4 Factor-Inwentash Faculty of Social Work, University of Toronto Graduate, Toronto, Ontario Canada; 5 Faculty Associate Member, Institute of Medical Science, University of Toronto, Toronto, Ontario Canada

**Keywords:** Children, Adverse childhood experiences, Fetal alcohol spectrum disorders, Comorbidity, Child protection, Criminal justice, Special education, Outcomes, Controls

## Abstract

**Background:**

Fetal alcohol spectrum disorder (FASD) is a highly prevalent lifelong disorder with high rates of comorbid neurodevelopmental disorders. Individuals with FASD are often exposed to abuse, neglect and foster home placements which have uncertain effects on the lifelong course of FASD. In this study we compare the prevalence of adverse childhood events (ACEs) and neurodevelopmental disorders in subjects with fetal alcohol spectrum disorders (FASD) and non-FASD controls.

**Methods:**

A cross-sectional chart review of patients referred to a regional developmental center was used to identify people with FASD and non-FASD controls. We recorded the number of ACEs and neurodevelopmental disorders in each patient’s chart. The most common diagnoses were attention deficit hyperactivity disorder, comprehension deficits, sleep disorders, and cognitive impairments. T-tests and a regression equation were utilized to determine significant differences between the groups.

**Results:**

The review identified 203 subjects, 98 with FASD and 105 non-FASD controls. Group mean age was 8.6 years and 64.5% were male. People with FASD were more likely to have any ACEs (mean 5.3) with ACE scores 3.7 points higher than non-FASD controls (mean 1.69) (t = 11.29; *p* < .001). Increased ACEs were associated with increased rates of neurodevelopmental disorders for people with FASD (R = .179, *p* = .026) but not for non-FASD controls (R = .130, *p* = .094).

Conclusions: Both FASD and subsequent exposure to ACEs are associated with increased risk for development of comorbid neurodevelopmental disorders. Prevention of ACEs during childhood may decrease risk for development of comorbid neurodevelopmental disorders.

## Background

In the United States, more than half of non-pregnant women of childbearing age reported drinking in the past month and one in five reported binge drinking [1]. Over 1 in 10 pregnant women (10.2%) self-reported alcohol use in the past month and 1 in 32 (3.1%) reported binge drinking in that same period [1].

Prenatal alcohol exposure increases an individual’s risk for a wide variety of disorders referred to collectively as Fetal Alcohol Spectrum Disorders (FASD) [2, 3]. FASD is a broad term encompassing a range of adverse effects including physical, mental, behavioral, and learning disabilities. Clinical diagnoses under this term have included Fetal Alcohol Syndrome (FAS), Partial Fetal Alcohol Syndrome, Fetal Alcohol Effect, Alcohol-Related Neurodevelopmental Disorder and Alcohol-Related Birth Defects [2, 4]. In the most recent revision of the Diagnostic and Statistical Manual of Mental Disorders a diagnostic category (Neurodevelopmental Disorder associated with Prenatal Alcohol Exposure) was included as an entity for further study [5]. In Canada, a consensus definition uses FASD a diagnostic entity [6].

In the United States, prevalence rates of FASD range from 2.4 to 4.8% among first-grade school-aged-children [7]. Among the United States annual live birth cohort of nearly 4 million, we could expect about 144,000 new cases of FASD each year (394 per day) using a midpoint prevalence rate of 3.6% [8]. FASD is commonly underdiagnosed in clinical settings limiting access to diagnosis-informed care for these people [9]. FASD also disproportionately affects people from disadvantaged families (poverty, low maternal education), children born to mothers with alcohol dependency, and children who are in foster/adoptive homes, orphanages, and institutions [2, 9].

FASD tends to become more complex across the lifespan. Increased prevalence of comorbid mental and developmental disorders among people with FASD has been an ongoing concern [3, 10–16]. When compared with prevalence estimates in the general population, people with FASD have increased rates of intellectual disabilities (22 times higher), anxiety disorders (11 times higher), psychosis (24.5 times higher), learning disabilities (2 times higher), attention-deficit/hyperactivity disorder (ADHD) (10 times higher), and oppositional defiant disorder (4.9 times higher) [16]. FASD is also increased in some indigenous populations [17].

One indicator of this increased risk is the prevalence of FASD in special sub-populations (e.g., children in foster care, special education, juvenile and adult corrections populations, and Indigenous populations). FASD rates in these populations are enormously high compared to the estimated global FASD prevalence in the general population of 7.7 per 1000 (95 CI: 4.9–11.7) [17]. FASD rates are increased 10 to 40 fold in foster care, special education, and juvenile and adult corrections [17]. Prevalence rates in foster care were 252 per 1000 (one in four children); in special education programs 82.0 per 1000 (1in every 12 students); and in psychiatric populations, the rate was 82.0 per 1000 (1 in every 12 patients). A study conducted in a secure forensic hospital found that 8% of patients met criteria for a diagnosis of FASD [18]. In a systematic review of prevalence in Canadian Correctional settings, prevalence ranged from 10.9 to 22.3% [19] The prevalence in an adult corrections system was 17.5% [20]. The authors reported that the rate could be as high as 31.2% if complete information was available. Prevalence of FASD are further increased among sub-populations where substance use disorders and mental health disorders are prevalent [21].

Mortality is an underappreciated aspect of FASD and often occurs in individuals before a diagnosis of FASD is made [22, 23]. Compared to the general population, the mortality risk for people with FASD is more than doubled and the mortality risk for siblings of people with FASD is increased 530% [24]. A diagnosis of FASD is also an important risk marker for maternal mortality. The maternal mortality rate in the 10-year period after birthing a child with FASD is about 4.5% and represents a 35-fold increase in mortality risk for birth mothers of a person with FASD [25]. Currently, there is very little information available regarding mortality risk for fathers of children with FASD.

FASD as a condition represents a high cost burden to service systems. The annual cost of care for children with FASD exceeds $23,000 and for adults with FASD, the cost exceeds $24,000 [26]. This study found that, compared to other common conditions, the estimated costs for children with FASD exceed those for autism ($23,000 versus $17,000, a 26% increase). The costs for adults with FASD exceeds those for diabetes ($24,000 versus $21,000 a 13.5% increase).

The expression of FASD has been conceptualized as a continuum of adverse outcomes from initial prenatal alcohol exposure to the increasing accumulation of adverse experiences [27, 28]. This suggests that a relationship between FASD and adverse childhood experiences (ACEs) may be important in understanding the confluence of events that impact individual outcomes over the lifespan.

Increasing rates of ACEs have been found to adversely impact adult health outcomes for exposed people [29]. ACEs are important markers for a wide range of health outcomes far into the future of the exposed child. Exposure to ACEs either individually, or more commonly as multiple exposures, increases risk for autoimmune disease, [30] multiple types of cancer, [31] and mental disorders in adults [32]. ACEs are also important risk markers for FASD since women with increased ACEs are at increased risk for alcohol use during pregnancy [33].

The relationship between ACEs and FASD has received very limited attention with only two published studies reporting on ACE scores in people with FASD. One study of 72 children referred for FASD assessment found very similar ACE scores in both the 47 children who received a diagnosis of FASD and the 25 who did not have FASD [34]. The total group mean ACE score was 3.4 (SD = 1.7), 1.5% of subjects had an ACE score of 0, 55.8% had an ACE of 1–3, and 42.6% had an ACE of 4–8 [34]. Another study examined the impact of neglect among children with an FASD and found that postnatal neglect did not have a detectable adverse effect on children with FASD [35]. This suggested that prenatal alcohol exposure leading to a diagnosis of FASD was an effect modifier on outcome independent of ACEs. However, the effects of postnatal neglect on outcome in this study were modest compared to the effects of prenatal alcohol exposure [35]. In addition to the ten traditional ACEs, two other adverse experiences are very common among children with FASD (foster care placement and residential care placement) [27, 36, 37].

In the current study, we compared patients with a diagnosis of FASD to non-FASD controls to investigate the relationships between i.) ACE scores and FASD diagnosis; and ii.) ACE scores and the number of comorbid neurodevelopmental disorders.

## Methods

We completed chart reviews of patients seen from 2010 to 2017 at the North Dakota FASD Center. This location serves as a regional referral center and patients are referred from a variety of sources: social services (30%), foster and adoptive parents (30%), physicians (30%), and schools (10%). The center evaluates about 200 new patients each year and follows a large number of children and adults with FASD.

Some of the subjects included in this study were also included in an analysis within a previously published paper on FASD and mental disorders [16].

Inclusion Criteria for FASD cases: We reviewed the charts of all patients under 22 years of age with any diagnosis under the FASD umbrella where FASD was diagnosed using the Fetal Alcohol Syndrome Checklist and the Alcohol Related Neurodevelopmental Disorder Checklist [38–40]. When the Alcohol Related Neurodevelopmental Disorder Checklist was added, we routinely began to collect data on ACEs. Charts and all available reports were reviewed for diagnosis of FASD, neurodevelopmental diagnoses, and ACEs.

Exclusion criteria for FASD cases: Any person over 21 years of age was excluded from the study. A few cases were referred with a diagnosis of FASD, but were excluded from the study since a history of prenatal alcohol exposure was not available. Individuals were also excluded if they did not have a diagnosis of any neurodevelopmental disorder (e.g. people assessed for speech or language delays - who did not have delays or children with bedtime problems).

Inclusion criteria for non-FASD controls: Patients were included if they were under 22 years of age, and did not meet criteria for any FASD and were seen between 2010 and 2017.

**Fig. 1:**
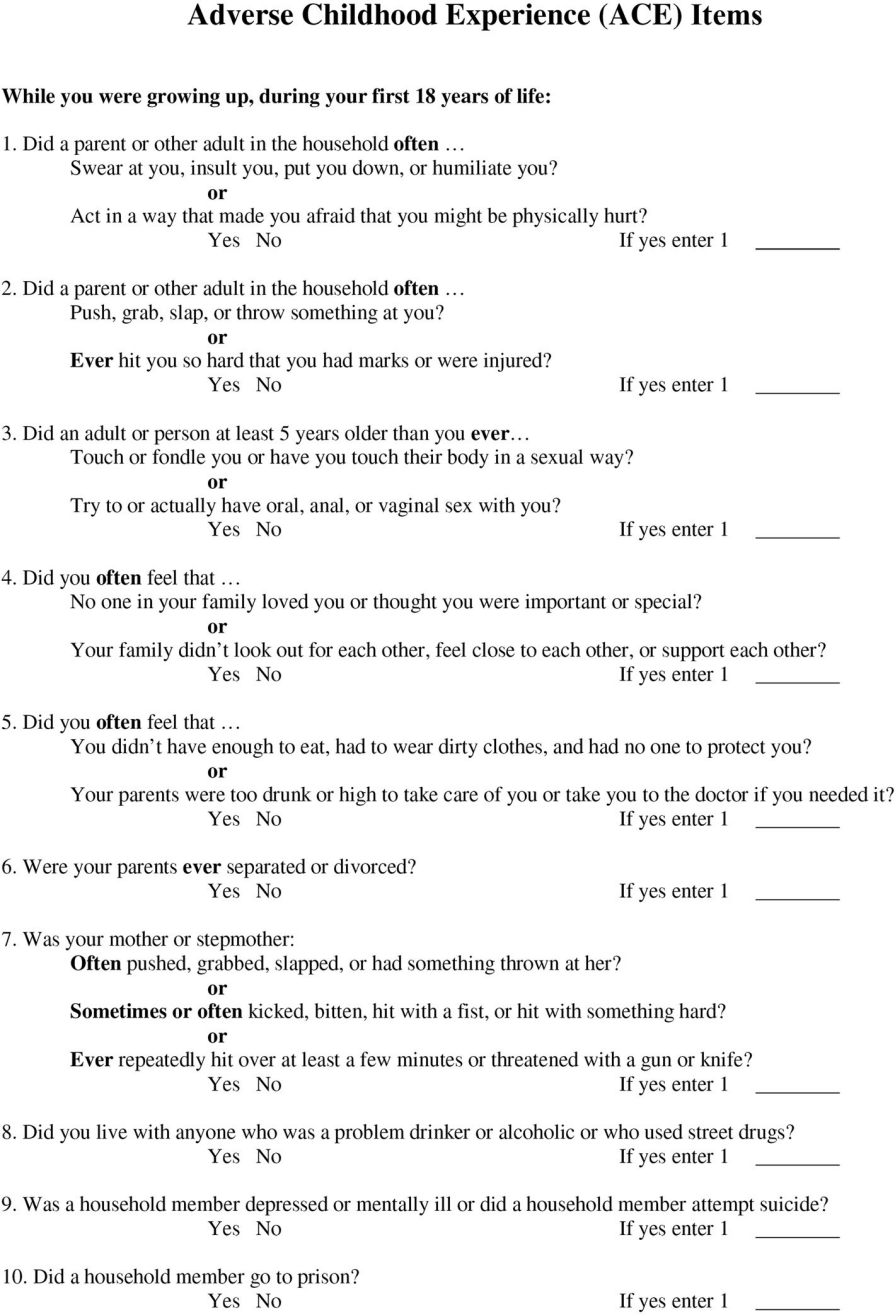
Title: Adverse Childhood Experience (ACE) Items Caption: The 10 adverse childhood experience items used in this study

We used the ten ACE items from the National Council of Juvenile and Family Court Judges to assess the prevalence of ACEs (Fig. 1) [41]. The score was comprised of ten binary variables, verbal/emotional abuse, physical abuse, sexual abuse, unloving family/emotional neglect, parental mental illness, neglect, parents divorced/separated, mother abused, drinking or drugs in house, and parent in prison.

We also captured two additional adverse events (placement in foster care and residential care), but these were not included in the ten item ACE score. ACE scores were obtained from social workers, parents, adoptive parents, foster parents, siblings, police reports, previous or current evaluations, medical charts, or interview. If an ACE score from a previous evaluation was included, we used that score. The 15 most common neurodevelopmental diagnoses (out of 25) routinely made at our center with a prevalence rate of more than 10% were subsequently used in the analyses as binary indicator variables. These included ADHD, oral comprehension deficits, sleep disorder, cognitive impairment (IQ < 85), visual impairment, anxiety disorder, speech disorder, enuresis, language disorder, memory impairments, and depression.

The diagnosis of neurodevelopmental disorders was included if the patient was previously diagnosed. Where applicable, the criteria from the Diagnostic and Statistical Manual of Mental Disorders were used [5]. Patients were diagnosed with input from genetics and dysmorphology, pediatricians, speech–language pathologists, ophthalmologists or optometrists, psychologists, neuropsychologists, psychiatrists, neurologists, pulmonology/sleep medicine, audiologists, nephrologists, otolaryngologists, educational diagnosticians, or as a part of a special education evaluation. If we disagreed with a previous diagnosis, we utilized the diagnosis from our center.

### Statistical analysis

The prevalence of the ACEs and the three ACE score groups were compared between subjects with FASD and the non-FASD controls using risk ratios. Average 10-item ACE scores were then compared between FASD and non-FASD groups and then for sub-groups of patients who had one of the 15 most prevalent comorbid diagnoses using one-tailed independent t-tests. Analysis of Covariance (ANCOVA) was used to examine the relationship between the ACE score and total number of diagnoses comparing FASD and non-FASD subjects. We also used the ANCOVA to test for interaction, indicating separate relationships between ACE score and total number of diagnoses for FASD patient compared to non-FASD. As interaction was significant, we examined the relationship between ACE score and total diagnoses by FASD and non-FASD groups using linear regression and Pearson’s correlation. A *p*-value < .05 was considered significant. No adjustments were made for repetitive tests.

## Results

We identified 728 cases eligible for chart review and identified 203 subjects who met our study inclusion criteria, including 98 patients with FASD and 105 non-FASD controls. For the total sample, the average age was 8.62 years (S.D. = 4.51) ranging from 2 to 20 years. The age distribution was: 31% aged 2 to 5 years; 37%, aged 6 to 10; and 32% were ages 11 to 20 years. In the total sample 131 (64.5%) were male.

Table 1 shows the prevalence of subjects with each ACE variable by group (FASD or non-FASD controls). Patients in the FASD group were more likely to have any of the 10 ACE items (*p* < .001) with the exception of parental mental illness (*p* = .810) (Table 1). Patients with FASD had significantly higher ACE scores (mean = 5.34, S.D. = 2.55) than non-FASD controls (mean = 1.69, S.D. = 2.01; t = 11.38, p < .001). The patients with FASD had the highest risk of living in a home with alcohol or drug use (RR = 4.96), being in foster care (RR = 9.05), having been neglected (RR = 6.73), and being exposed to an unloving family/emotional neglect (RR = 3.39). Children with FASD were over three times as likely to have ACE scores from 2 to 5 and over 6 times more likely to have ACE scores from 6 to 10. This relationship was not observed for the non-FASD controls (Table 1). A diagnosis of FASD was found to increase the risk for ACEs, demonstrating that the adverse impact of FASD is apparent early in life and is a persistent risk marker for exposure to childhood adversity.

**Table 1:** Prevalence of ten ACE items for children with and without FASD

ACE	FASD		Non-FASD		RR	p
	N	%	N	%		
Parents Divorced/Separated	71	72.45	48	45.71	1.86	<.001
Drinking/Drugs in Home	83	84.69	24	22.86	4.96	<.001
Neglect	85	86.73	15	14.29	6.73	<.001
Unloving Family	67	68.37	12	11.43	3.39	<.001
Depression	32	32.65	37	35.24	0.94	.810
Physical Abuse	49	50.00	10	9.52	2.44	<.001
Verbal Abuse	46	46.94	8	7.62	2.44	<.001
In Prison	35	35.71	8	7.62	2.07	<.001
Mother Abused	32	32.65	9	8.57	1.92	<.001
Sexual Abuse	23	23.47	6	5.71	1.84	<.001
*In Foster Care*	*89*	*90.82*	*17*	*16.19*	*9.05*	*<.001*
*In Residential Care*	*19*	*19.39*	*3*	*2.86*	*1.98*	*<.001*
None or One	10	10.20	61	58.10		
Two to Five	35	35.71	37	35.24	3.45	<.001
Six to Ten	53	54.08	7	6.67	6.27	<.001

In residential or foster care not included in total ten-item ACE score

The relationship between FASD and ACEs was associated with increased risk for the development of additional neurodevelopmental disorders (Table 2). Among subjects with FASD, the most prevalent diagnoses were ADHD (85.7%), oral comprehension deficits (75.5%), sleep disturbance (63.3%), cognitive impairment (IQ < 85) (61.2%), and visual impairment (53.1%) The number of comorbid diagnoses in this study ranged from 0 to 11 (mean = 5.17, S.D. = 2.24). These were higher on average for patients with FASD (mean = 5.98, S.D. = 2.10) than controls (mean = 4.42, S.D. = 2.12, t = 5.27, *p* < .001). Mean ACE scores were also significantly higher for patients with FASD who had any one of the 15 neurodevelopmental diagnoses (*p* < .01) (Table 2). Patients with FASD who had multiple neurodevelopmental diagnoses also had increased ACE scores (all p < .01).

**Table 2:** Average 10-item ACE scores for children with common eleven comorbid diagnoses between FASD and non-FASD children

11 Most Common Diagnoses	FASD			Non-FASD			t	p
	N	Mean	S.D.	n	Mean	S.D.		
ADHD	84	5.36	2.60	67	1.78	2.12	9.12	<.001
Oral Comprehension	74	5.53	2.47	53	1.55	1.95	9.76	<.001
Sleep Disorder	62	5.56	2.56	55	1.80	2.26	8.39	<.001
Cognitive Impairment	60	4.85	2.58	32	1.87	2.25	5.49	<.001
Vision Problems	52	5.63	2.63	29	1.48	1.92	7.46	<.001
Anxiety Disorder	40	5.75	2.71	40	1.48	1.97	8.07	<.001
Speech Difficulties	42	5.50	2.50	38	1.55	1.86	7.95	<.001
Enuresis	26	5.11	2.83	23	1.48	1.53	5.48	<.001
Language Problems	22	5.55	2.79	24	1.29	1.81	6.19	<.001
Memory	31	6.00	2.48	7	1.71	1.98	4.27	<.001
Depression	22	5.86	2.27	14	1.79	2.01	5.48	<.001
Learning Disability	12	5.17	3.27	13	1.85	1.86	3.15	.004
Autism	4	5.00	1.41	20	0.90	1.83	4.20	<.001
Intellectual Disability	10	5.30	2.21	10	1.10	2.18	4.27	<.001
Hearing Disorder	10	5.50	2.59	10	0.90	0.88	5.32	<.001
Total Diagnoses								
0 to 3 Dx	12	4.75	2.63	36	2.11	2.28	3.34	.002
4 to 6 Dx	46	5.13	2.42	49	1.20	1.57	9.45	<.001
7 to 11 Dx	40	5.75	2.67	20	2.10	2.27	5.24	<.001

Other Diagnoses not shown: Verbal/Performance Discrepancy (≥ 15 points), Tremors, Tics, Tourette’s Syndrome, Obsessive Compulsive Disorder, Encopresis, Cerebral Palsy, Alcohol Abuse, Substance Abuse, and Tobacco Use

**Fig. 2:**
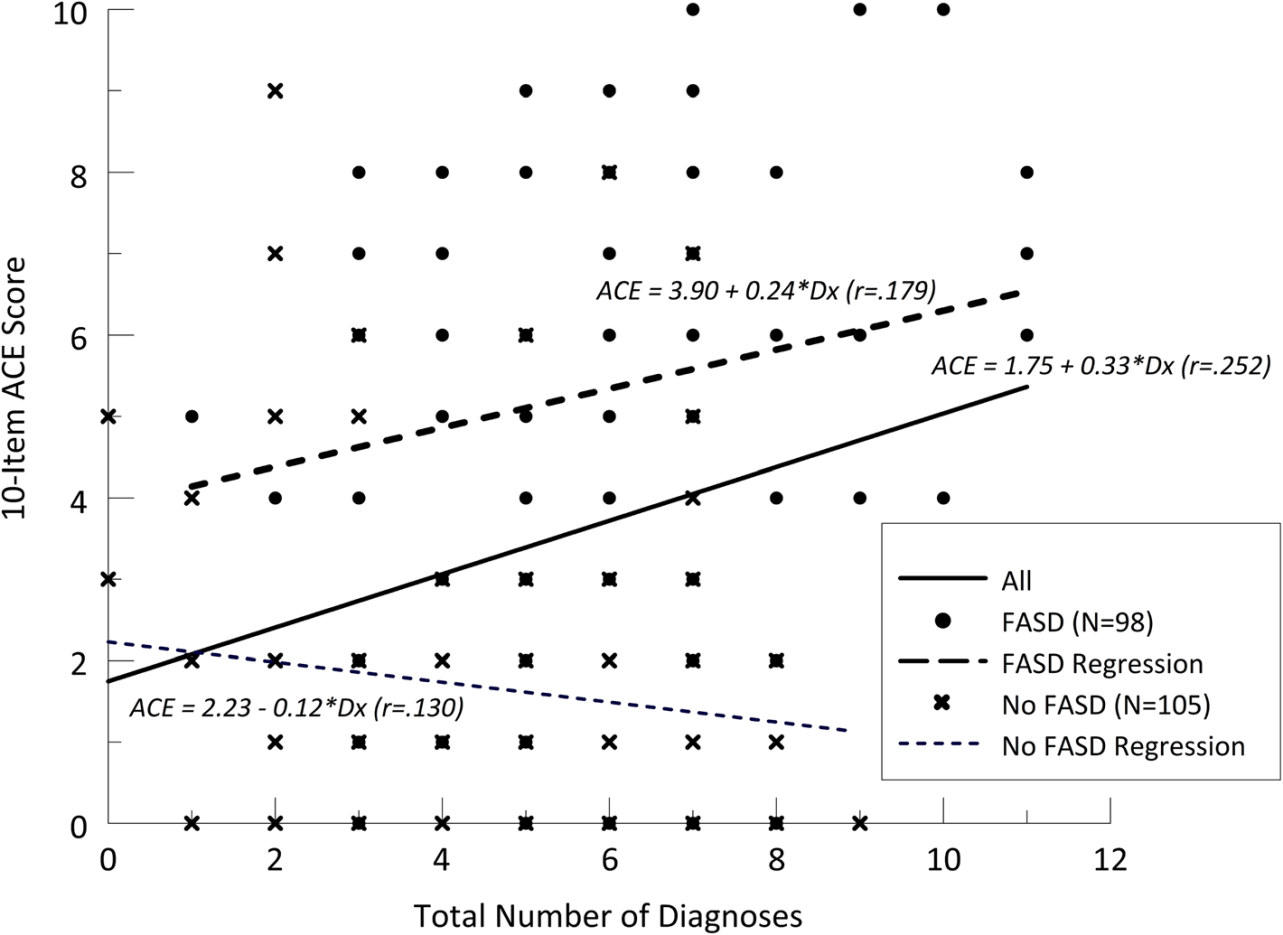
Title: Depiction of Relationships Between Fetal Alcohol Syndrome Disorders (FASD), adverse childhood experiences (ACEs), and Neurodevelopmental Disorders Caption: Prediction of 10 ACE scores from total number of diagnosis for children with FASD (*n* = 98) and non-FASD controls (*n* = 105)

The patients with the highest rates of ACEs (Table 1) and neurodevelopmental disorders (Table 2) were primarily from the FASD group. An analysis of covariance (ANCOVA) was used to estimate the relationship between ACE scores and total neurodevelopmental diagnoses for children with FASD compared to non-FASD controls. The ANCOVA demonstrated highly significant interaction (F = 5.74, *p* = .017) between the number of diagnoses and FASD when predicting the ACE score. The data were then split between FASD or non-FASD controls and independent regressions were calculated on total diagnoses and the ACE score (Fig. 2). The relationship between ACE score and total number of diagnoses was significant for the total group (*n* = 203) (R = .179, *p* < .001) and for the 98 people with FASD (R = .252, *p* = .026). This relationship was not significant for the 105 people without FASD (R = .130, *p* = .094).

**Fig. 3:**
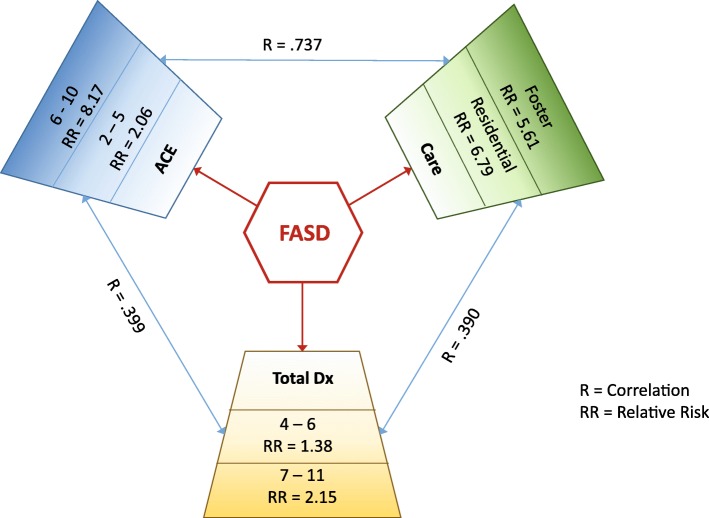
Title: Relationship Between FASD, ACES and Neurodevelopmental comorbidity. Caption: Association between FASD, ACES and neurodevelopmental disorders

In Fig. 3 we illustrate the relationship between FASD, ACEs, and neurodevelopmental disorders. FASD is associated with an increased ACE score. A person with FASD has twice the risk of having an ACE score from 2 to 6 when compared to a person without FASD (RR = 2.06). The risk for an ACE score of 7 or more is increased over 8 times (RR = 8.16). FASD is also associated with a large increase in placement in foster care or residential care. People with FASD have over fivefold risk of foster care placement and nearly a 7-fold increase in risk for placement in a residential care facility. Increasing ACE scores were correlated with increasing risk for foster care or residential care placement. FASD is also associated with increased risk of a neurodevelopmental diagnoses. A person with FASD has nearly twice the risk of having 5–7 comorbid neurodevelopmental disorders (RR = 1.38) and over twice the risk to have 8 to 15 comorbid diagnoses (RR = 2.15).

## Discussion

In this study we demonstrate that ACEs are far more common in people with FASD compared to non-FASD controls. Only 6.1% of subjects with FASD had very low ACE scores (0 or 1) compared to 58.1% of non-FASD controls (difference of 950%). Conversely, only 6.7% of controls had very high ACE scores (7–10) compared to 54% of children with FASD (an 805% increase). People with FASD had an average of 3.7 more ACEs than controls. Among subjects with FASD the most prevalent ACEs were: neglect (87%), parental substance abuse (85%), parental separation or divorce (50%) and physical abuse (50%). These data support the concept that in affected individuals, FASD symptomatology increases in severity over time. In part, this is due to postnatal exposure to adversity, which is potentially preventable. While several other studies have also identified a strong relationship between FASD and increased risk for comorbid mental disorders and developmental disabilities, those studies did not examine the important effect of vulnerability for ACEs in people with FASD [3, 14, 16, 36].

ACEs are well known to be associated with increased risk for multiple adverse health outcomes over a person’s lifespan [29, 42]. In this study, we demonstrate an important relationship between FASD, postnatal adversity (ACEs) and increasing rates of comorbid neurodevelopmental disorders. AFASD appears to be an especially important risk factor for the development of neurodevelopmental comorbidity. This has important influences on both severity of FASD and burden of care. The increasing rates of comorbid neurodevelopmental disorders in FASD creates a huge service demand on foster parents, birth parents, schools, and the mental health systems of care. This study offers some explanation as to why FASD is so costly to service systems when compared to other conditions such as autism spectrum disorder [26].

Currently, our understanding of the full range of the expression of FASD across the lifespan is significantly limited by a scarcity of studies in both adult and geriatric populations of affected individuals [27]. Improved understanding of FASD symptomatology and experiences over the lifespan are important since nearly all specialists in medicine and allied health services are likely to treat people with FASD in their care settings [43].

A frequent clinical concern is how to improve our understanding of how a diagnosis of FASD should change routine clinical care compared to people with other neurodevelopmental disorders or people who do not have FASD. The current study presents a compelling case for early diagnosis and specialized treatment plans for children with FASD [16, 25, 40]. The rates of potentially preventable problems in people with FASD are compelling. Children with FASD have a 9-fold increase in risk for foster care placement, and a 19-fold increase in risk for contact with juvenile corrections services [37]. The multidisciplinary teams caring for these children will need to have extensive resources and training to care for children with this level of complexity. This should include modifying treatment plans to include prevention of conditions such as ACEs.

FASD also imposes huge workloads and service demands on social services, child protection services, the foster care systems, and dependency courts. Children with FASD are 9 times more likely to be placed in foster care and are 6.7 times more likely to be placed in residential care (Table 1). One in five children placed in residential care have multiple foster home placements prior to their current placement, which was primarily for severe neurobehavioral disorders. Both foster and residential care services are costly and are often viewed by involved children as being stressful and traumatic.

This study demonstrates the importance of increased involvement of pediatricians and child and adolescent psychiatrists/psychologists to improve early identification of FASD. Pediatricians have the opportunity to be leaders in the effort to implement early identification of neurodevelopmental conditions and to provide early preventative or intervention services. FASD is an example of a prototypic condition for early diagnosis with a vast amount of potential for prevention of ACEs and secondary disabilities.

This study has several limitations that should be considered in interpreting this data. FASD services vary across clinical sites with respect to diagnostic capacity and guidelines.(38,44) As a result, the population of patients diagnosed with FASD at our center may differ modestly compared to other sites [38, 44]. However, several studies have been conducted to compare the utility and accuracy of diagnostic criteria for FASD across multiple centers. These studies found reasonably similar rates of agreement between the different diagnostic schema. The patient population seen at the North Dakota FASD Center may also differ from other patient populations, such as those seen at large urban centers. Other clinical sites may also see patients from different ethnic or racial sub-populations which could modify the rates of ACEs and diagnosis of neurodevelopmental disorders. Most of the patients seen at our center had prenatal polysubstance exposure which may differ from patterns of exposure in other centers.

Further research on strategies to prevent neurodevelopmental disorder comorbidity over the lifespan is urgently needed. FASD prevention should focus on identification of before and during pregnancy. Early identification and diagnosis of FASD in young children has the additional benefit of identifying a mother who is more than 70% likely to have another affected child if she continues to consume alcohol during subsequent pregnancies [45]. Anticipatory guidance should also emphasize the link between prenatal alcohol exposure leading to FASD, ACEs, and secondary disabilities including: school failure, substance abuse, multiple foster home placements, peer exploitation, incarceration, and premature death. These secondary disabilities occur across the lifespan for many people with FASD and are potentially preventable conditions resulting from inadequate screening, inaccessible diagnostic and support services, and lack of access to, or underutilization of diagnosis informed care. As an example, early diagnosis could lead to implementation of strategies to prevent postnatal adversity (ACEs) and would represent a potential benefit of diagnosis informed care in people with FASD.

Early diagnosis and management of FASD in affected individuals may offer a path to prevention of many of these comorbid secondary disabilities. Improved screening strategies are now emerging and may provide an opportunity to improve outcomes via early access to intervention [40]. Optimal screening strategies will need to be paired with huge improvements in access to community-based services for early diagnosis with an emphasis on detection of prenatal alcohol exposure, fetal alcohol spectrum disorder, development disorders, and mental disorders.

## Conclusions

The prevalence of ACEs in people with FASD is greatly increased compared to people without FASD. Importantly, ACEs are postnatal events that can potentially be prevented which could reduce symptom severity and decrease demands on service systems which could also reduce costs.

Lastly, the relationship between FASD and ACEs in this study demonstrated that children with FASD were 9 times more likely to be in foster care and 6.7 times more likely to be placed in residential care compared to controls. Further research is needed to determine if prevention of ACEs decreases risk for neurodevelopmental disorders and additional adversity across the lifespan.

## Data Availability

The dataset used and/or analyzed during the current study is available from the corresponding author on reasonable request.

## Abbreviations

ACE: Adverse childhood experience
ADHD: Attention deficit hyperactivity disorder
Dx: Diagnosis
FASD: Fetal alcohol spectrum disorder
RR: Relative risk
S.D.: Standard deviation
